# Thriving at work as a mediator between nurses’ structural empowerment and job performance, work-personal life benefits, stress symptoms and turnover intentions: a cross-sectional study

**DOI:** 10.1186/s12912-025-02828-0

**Published:** 2025-02-14

**Authors:** Maria Engström, Annica Björkman, Marit Silén, Anna Carin Wahlberg, Bernice Skytt

**Affiliations:** 1 Department of Caring Science, Faculty of Health and Occupational Studies, University of Gävle, Kungsbäcksvägen 47, Gävle, 801 76 Sweden; 2 Medicine College, Lishui University, No. 1 Xueyuan Road, Lishui City, China; 3 Department of Neurobiology, Care Sciences and Society, Karolinska Institutet, Stockholm, 171 77 Sweden

**Keywords:** Intention to leave, Nursing, Quality of care, Structural empowerment, Stress, Thriving, Working conditions, Working life, Work-personal life benefits

## Abstract

**Background:**

Nurses work in a knowledge-intensive sector with high demands for lifelong learning. Thriving is a positive psychological state, including a sense of mutual learning and vitality at work. Research on thriving, its antecedents and outcomes is called for*.* The study aim was to examine thriving as a mediator in the relationships between telephone nurses’ structural empowerment and the outcomes work-personal life benefits, job performance, work-related stress symptoms and turnover intentions, as well as to psychometrically test the Thriving Scale (Swedish version).

**Methods:**

Questionnaire data, a national sample of 409 Swedish telephone nurses, were collected, and relationships were examined using multiple regression analyses with PROCESS macro. Factorial validity of the Thriving Scale was tested using confirmative factor analyses.

**Results:**

There were statistically significant relationships between structural empowerment and the outcomes (work-personal life benefits, job performance, stress symptoms, turnover intentions), and these relationships were mediated by thriving. The Thriving Scale showed good internal consistency, and an acceptable to borderline mediocre fit for factorial validity. Thirty-two percent reported turnover intentions.

**Conclusions:**

Good access to structural empowerment increases nurses’ thriving, which in turn improves work-personal life benefits, job performance, and decreases stress symptoms and turnover intentions. Managers should strive to improve nurses’ thriving at work, emphasizing good access to empowering structures.

**Supplementary Information:**

The online version contains supplementary material available at 10.1186/s12912-025-02828-0.

## Background

In Spreitzer’s description of “A socially embedded model of thriving at work”, thriving is described as both a sense of vitality and a sense of learning for the employee at work [1]. Thriving, in turn, has been found related to outcomes such as employee health, wellbeing, job performance, attitudes, and development, according to an integrative review [2]. The described antecedents of thriving are, for example, job demands and resources, training opportunities at work, recognition, support and trust in workplace relationships [2]. Reviews of thriving have called for more research on the relationships between thriving and positive health outcomes such as benefits for personal life [2], and research on more complex models involving antecedents of thriving, thriving and its outcomes [3, 4]. Within nursing, the research on thriving is limited, but there has been an upward trend during recent years. Still, nursing is a profession within a knowledge-intensive sector that requires lifelong learning and where stress [5], turnover and turnover intentions are quite common [6, 7]. Thus, the focus here was on increasing our knowledge about thriving at work among registered nurses in Sweden working in telephone nursing, and on studying thriving as a mediator between telephone nurses’ structural empowerment and positive outcomes such as job performance/nurse satisfaction with given care, work-personal life benefits as well as negative outcomes such as stress symptoms and turnover intentions. Furthermore, to psychometrically test the Thriving Scale (Swedish version).

### Telephone nursing

Telephone nursing in the Swedish Healthcare Direct means working alone but with your colleagues nearby in a traditional call centre [8]. The nurses are also allowed to work from home after agreement with the manager in charge. To ensure consistency and enhance patient safety the nurses are obliged to use a decision support system and structure the conversation according to the internally developed conversation model Dialogue Process [9]. The service is open all hours and every day of the week. Challenges described are e.g., demanding work, cognitive fatigue, high employee turnover, lack of possibilities to discuss, reflect and socialize with colleagues [10]. The work has been described as lonely and stressful with high responsibility but also as a stimulating work [10, 11]. Stress, fatigue and understaffing, in turn, have been described in interviews as contributing factors of malpractice claims [12]. Good prerequisites in telephone nurses work c.f., empowering structures have been described as central for their performance and wellbeing [10, 11].

### Theoretical framework: Kanter’s theory of structural empowerment and Spreitzer’s model of thriving at work

According to *Kanter’s theory of structural empowerment* [13], the essential conditions promoting staff wellbeing and effectiveness are access to information, support, resources and development opportunities as well as formal and informal power. The staff need good access to the information required for work and the unit’s goals. Furthermore, support in and feedback about work, resources, e.g., equipment to perform work, time and opportunities to develop at work. According to the theory, these conditions are influenced by staff members’ informal power (networks/relationships of importance to work) and formal power (a central and visible job within the organization). The concepts in Kanter’s theory can be seen as contextual factors of importance for staff wellbeing and performance. Extensive research within nursing has also provided support for the link between structural empowerment and staff wellbeing, e.g., stress symptoms, emotional exhaustion and burnout [14–16], work-life balance [17], job satisfaction [18–20], staff turnover and turnover intentions [6, 21]. Furthermore, there is support for a link between structural empowerment and work effectiveness, e.g., evidence-based work, staff-rated care quality e.g., [14, 22], person-centred care processes [23], nurse professional competence [24], and patient-reported satisfaction with care [25]. Regarding thriving, structural empowerment has also been found related to thriving [23, 24]. Kanter views structural empowerment as the base for staff members wellbeing and performance and even more important than personal factors [13]. The connection to thriving is based on that contextual factors (c.f., structural empowerment) have been found related to thriving, this is also in line with *Spreitzer’s model “A socially embedded model of thriving at work”* [1] and reviews of thriving [2, 3]. Several of the reported antecedents of thriving have similarities with the dimensions of structural empowerment. These reported antecedents include broad information sharing, knowledge resources such as access to information, a climate of trust and respect, relational resources such as connectivity with others at work [1], perceived organizational support [3], training opportunities, recognition, support, and resources [2]. A systematic review and meta-analyses [3] showed that thriving was associated with several relational aspects, such as supportive and empowering leaders and supportive co-workers, trusting relationships, workplace civility and organizational support, and the outcomes of thriving were, e.g., self-assessed burnout, job performance, work commitment, job satisfaction, turnover intentions and health. Similar outcomes are described in other reviews [2, 4] and in the socially embedded model of thriving at work [1].

Within nursing, structural empowerment [23, 24] and person-centred care processes [23], as well as organizational practices such as Lean management [26] and organizational intelligence [27] have been found related to thriving. Furthermore, thriving has been found negatively related to workplace violence [28, 29], work-family conflicts [30] and positively to authentic leadership [28, 31], family supportive supervisors [30], psychological capital, workplace mindfulness, organization justice [28], years of experience [28, 32] and perceived organizational support [28, 33]. Personal variables such as age and educational level were linked to thriving in one study [27] but not in another one [28]. Regarding outcomes, thriving has, in nursing, been found to be positively related to job satisfaction [29, 34, 35], organizational commitment [35], work performance [32] and psychological empowerment [23], and negatively related to turnover intentions [29]. However, increased learning has been shown to be related to increased odds of leaving the profession for registered nurses (vs. leaving the unit) when the other variables in the model (vitality, age and resources) were held constant [6]. Thus, emphasizing the importance of increasing both learning and thriving as learning without a sense of vitality hampers thriving, as does vitality without learning [36].

### Thriving as a mediator

Research on thriving as a mediator between contextual factors and outcomes has been conducted to a lesser extent. Within nursing research, we found two studies [32, 34] of thriving as a mediator. Jiang et al. revealed that thriving mediated the relationship between task identity or autonomy and job satisfaction among 892 nursing students under clinical practice education in Australia. In addition, they showed that the relationship was moderated by students’ rating of their mentor’s mentorship (supported for task identity, but not for autonomy) [34]. Shen et al., [32] found thriving as a mediator between psychological resilience and work performance among 308 clinical nurses in China. Their results showed that both psychological resilience and thriving had a significant positive effect on work performance. Others, not within healthcare, have looked at outcomes such as life satisfaction [37], job performance [38, 39], and organizational commitment [40]. If thriving is related to both job and life satisfaction, it could be assumed that there is also a link to work-personal life benefits. Only two of the above-mentioned mediation studies were carried out within nursing [32, 34]. Of the above studies Abid et al., [40] used a two-wave time lagged design and found that contextual factors and thriving at time 1 were related to organizational commitment measured one month later. Furthermore, there was a direct effect between contextual factors and organizational commitment and an indirect effect through the mediator thriving [40]. Two other studies also investigated thriving as a mediator between contextual factors and life satisfaction [37] and job satisfaction [34]. Both studies found that contextual factors measured at time 1 were related to thriving measured at time 2 and life satisfaction measured at time 2 and in the study by Jiang et al., [34] with job satisfaction measured at time 3. The results confirmed thriving as a mediator between contextual factors and life or job satisfaction [34, 37]. Elahi et al., [38] also used a time lagged study and staff performance was measured later using responses from immediate managers. Frazier and Tupper [39] also measured the outcome job performance in a similar way using immediate managers. In both studies thriving was found as a mediator between contextual factors and job performance.

To sum up, there has thus far been limited research on thriving within nursing, and one integrative review of thriving within different areas concluded that there is a need for more research on the outcomes of thriving, especially positive outcomes but also outcomes that offer benefits beyond the workplace, such as work-life balance [2]. There is furthermore a need for more complex models of relationships concerning thriving, its antecedents, and outcomes [3, 4].

Thus, the aims of the present study were to examine thriving as a mediator in the relationships between telephone nurses’ structural empowerment and positive outcomes, such as work-personal life benefits and job performance (satisfaction with given care), as well as negative outcomes, such as work-related stress symptoms and turnover intentions. A further aim was to psychometrically test the factorial validity and internal consistency of the Thriving Scale (Swedish version).

**Fig. 1:**
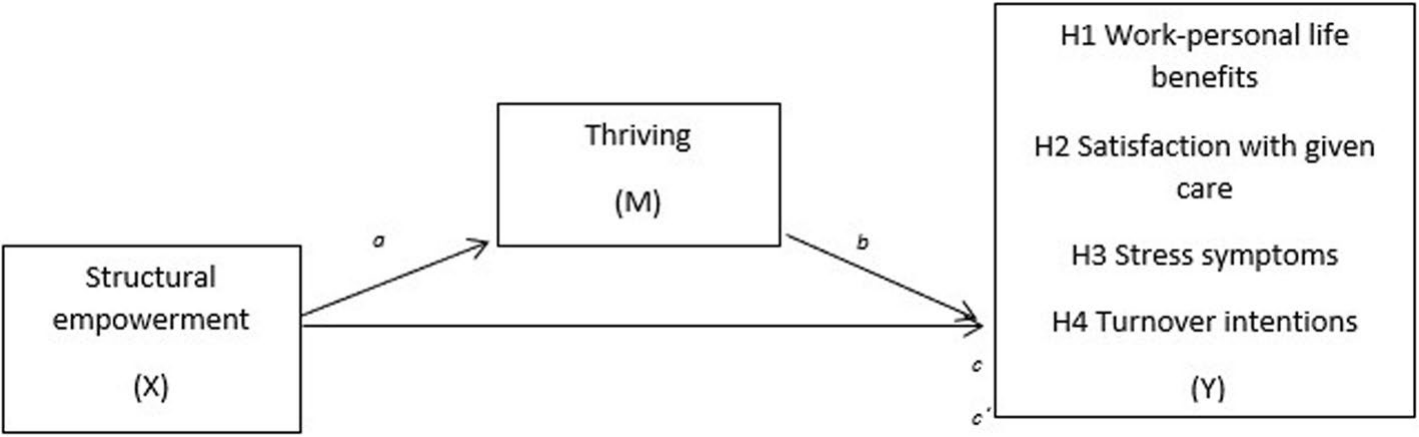
An illustration of the models tested, one for each hypothesis. The coefficient *a* represents the effect of Structural Empowerment (X) on the Mediator Thriving. The coefficient *b* is the effect of M on Y (Work-personal life benefits OR Satisfaction with given care OR Stress symptoms OR Turnover intentions) controlling for X. The coefficient *c* is the direct effect between X and Y, and *c’* is the effect of X on Y while controlling for M

The hypotheses (H) based on the literature were:

- H1a, there is a positive association between structural empowerment and work-personal life benefits, and H1b, the association is mediated by thriving.
- H2a, there is a positive association between structural empowerment and satisfaction with given care, and H2b, the association is mediated by thriving.
- H3a, higher structural empowerment scores are related to fewer stress symptoms, and H3b, the association is mediated by thriving.
- H4a, there is a negative association between structural empowerment and turnover intentions, and H4b, the association is mediated by thriving (Fig. 1).

## Methods

### Design, sample and setting

A cross-sectional correlational design was applied. A national sample of 859 telephone nurses were invited and 409 responded to a questionnaire in 2017 about working life (response rate 47.6%). The telephone nurses worked at the 23 sites of national healthcare service, Swedish Healthcare Direct. The Swedish Healthcare Direct is a nationwide healthcare advice service open 24/7 all year round, handling approximately 5.5 million calls a year. The nurses work at a traditional call centre. To assist in their assessment of care seekers, they use an established telephone nursing dialogue process along with a compulsory computerized decision support system for triage support and documentation. The computer system used also shows the number of calls waiting in the telephone queue, mean waiting time, and the number of colleagues, e.g., other telephone nurses logged in and active within Swedish Healthcare Direct.

### Data collection

A web-based survey was sent in 2017 using Sunet Survey (a Swedish survey platform) and two reminders were sent to non-responders. The 19-item Condition of Work Effectiveness Questionnaire CWEQ-II [41], Swedish version [14] was used to measure structural empowerment. The instrument consists of six factors: opportunities, information, support, resources, formal power and informal power. Response alternatives are 5-grade (1. None to 5. A lot). For factor scores, the items are averaged. Total scale is used in the present study (sum of factor scores), and higher scores represent greater structural empowerment; 6 to 13 designate low levels of structural empowerment, 14 to 22 moderate levels and 23 to 30 high levels. Cronbach’s Alpha [α] in the present study 0.89. Work-personal life benefits were measured using one item about to what extent working life affects private life positively, with response alternatives from 1) a very small degree to 5) a very high degree [42]. Work-related stress symptoms were measured using the factor stress symptoms from the Psychosomatic Health Aspects Scale (α 0.84) [43]. Response alternatives are 5-grade [1) very often, 2) Quite often 3) Sometimes 4) Seldom 5) Never]. For factor scores, the ten item scores are averaged, and higher scores represent fewer stress symptoms. Nurses’ job performance was measured using the 8-item Nurse Satisfaction with Given Care Scale (α 0.91) [44]. Response alternatives are 7-grade Likert scale (1. Not at all to 7. To a very high extent). For total score, the item scores are averaged, with higher scores representing greater satisfaction. The thriving scale [36, 45] was used to measure thriving. The scale consists of 10 items (α 0.90), two factors: learning (α 0.84) and vitality (α 0.87). Response alternatives are 7-grade Likert scale (1. Disagree strongly to 7. Agree strongly). For factor scores and total score, the corresponding items are averaged, with higher scores representing greater thriving. Turnover intention was measured using one item ‘Have you recently seriously considered quitting your job on the unit because you don’t enjoy it?’, with response alternatives 1) No, 2) Yes, but I have not done anything and 3) Yes, and I have taken action [43]. All instruments used (CWEQ-II, Thriving Scale, Stress Symptoms, Nurse Satisfaction with Given Care) have Swedish versions and some were developed in Sweden. All have shown good psychometric properties in previous studies; see references above. The Swedish version of the Thriving Scale has not previously been tested for factorial validity, thus conducting such a test was one of the aims of the present study.

### Data analysis

Data were analysed using IBM SPSS Statistics 27 and the macro PROCESS v4.0 for the regression and mediation analyses. We performed mediation analyses with PROCESS macro model 4. Strengths with this approach are that 95% confidence intervals of the indirect effect are calculated using bootstrapping test which is more likely to be accurate than when the normal theory approach is used and has higher power compared to for example Sobel test [46]. Dependent variables were work-personal life benefits, satisfaction with given care, stress symptoms and turnover intentions. Predictors were structural empowerment and thriving (mediator), see Fig. 1. Age and sex were included in the models as control variables and kept in the final model if statistically significant. Sex was non-significant in all four models. Age was statistically significant in the models of stress symptoms and turnover intentions but not for nurse satisfaction with care and work-personal life benefits. The analyses were run with and without outliers. Analyses with outliers are presented in the results section. When the results differed between analyses with and without outliers with respect to statistically significant and non-significant results, this is commented on in the text, and the results of both analyses are presented in the supplementary file. If missing values were 10% or less in a factor or scale, it was replaced with the individual’s mean value in that factor/scale. If more than 10% of the items in a scale or in a factor were missing the individual was treated as missing in that scale/factor. IBM AMOS was used for the confirmative factor analysis (CFA) of the thriving scale. Pearson’s correlation coefficient (r), multiple linear regression analyses and binary logistic regressions were used to study relationships between variables. Multicollinearity was tested with variance inflation factor (VIF) and values were below 2.5, indicating no problems. Turnover intention was dichotomized to ‘yes’ and ‘no’ and analysed using student t-test, effect size (ES) with Cohen’s d, and binary logistic regressions. According to Cohen [47], an ES of 0.20 could be seen as small, 0.50 as medium and 0.80 as large. For the mediation analyses, bias corrected 95% confidence intervals (CIs) with 5000 bootstraps are presented. Goodness-of-fit indices used were model Chi^2^, root mean square error of approximation (RMSEA), comparative fit index (CFI), and Tucker-Lewis index (TLI). Descriptive data presented are means, standard deviations (SDs) and CIs. Statistical significance was set at *p* ≤ 0.05. To test for potential risk of common method variance we performed Harman’s single factor test. All items of the instruments included in each model were added in an explorative factor analysis. The variance explained by the first factors ranged from 28.8% to 33.8% and all had several factors with eigenvalues more than 1, thus, indicating low risk of common method bias [48].

### Ethical considerations

The regional ethical board approved the study (Ref. no. 2014/409). All participants received written study information. Answering and returning the web-survey were regarded as their tacit informed consent.

## Results

### Thriving Scale (Swedish version), factorial validity and internal consistency

The fit indices for the two-factor second-order CFA model of thriving revealed a significant Chi^2^ (161.76; df 33;*p* < 0.001). However, CFI was 0.951, TLI 0.918 and RMSEA 0.098 (90% CIs 0.083 to 0.113), thus indicating acceptable support for the factorial validity of the Swedish version of the Thriving Scale with regards to CFI and TLI. Cutoff close to 0.95 is recommended by Hu and Bentler [49] and Zyphur [50] for CFI and TLI, while Bentler and Bonnet [51] recommend 0.90 or more for TLI. However, for the RMSEA the results indicate mediocre fit with a value between 0.08 to 0.10 [50, 52]. The standardized regression weights for the factors learning and vitality ranged from 0.708 to 0.926 except for two items ‘… not learning’ 0.353 and ‘don’t feel very energetic’ 0.419, see Supplementary file 1. Cronbach’s Alpha (α) was 0.90 for the total scale, and 0.84 and 0.87 for the factors learning and vitality, respectively.

### Descriptive values of the study variables, bivariate correlations (r) and comparisons

Most participants were female (96.3% of 409) and worked part-time (70.9%); their mean age was 55.4 years (SD 9.1). Mean values, SDs, α-values and associations between the study variables are presented in Table 1. There were statistically significant positive relationships between thriving, its factors and all other study variables. Note that higher scores on the stress symptoms factor represent fewer stress symptoms. Furthermore, there were statistically significant positive relationships between structural empowerment and all other study variables. Comparing the two turnover intention groups, ‘no’ and ‘yes’, the results revealed statistically significant differences for all study variables (*p* < 0.001) except for nurse satisfaction with given care (*p* = 0.165), with ES 1.0 for structural empowerment and 1.3 for thriving (learning 1.0 and vitality 1.3). The group with no turnover intentions reported higher scores than the group with turnover intentions for thriving, the factors learning and vitality, structural empowerment, work-personal life benefits and stress symptoms (i.e., less stress symptoms) (Table 2). In the group turnover intention yes, 52 reported ‘Yes, and I have taken action’ and 79 ‘Yes, but I have not done anything’.

**Table 1:** Study variables, descriptive statistics and bivariate correlations

	**Mean (SD)**	**α**	**Age**	**Thriving**	**Learning**	**Vitality**	**SE**	**W-PLB**	**NSC**
**Thriving**	5.2 (1.0)	0.90	−0.03						
• **Learning**	5.5 (1.0)	0.84	−0.07						
• **Vitality**	5.0 (1.1)	0.87	0.04		0.68***				
**Structural empowerment**	18.8 (3.8)	0.89	−0.04	0.63***	0.60***	0.56***			
**Work-personal life benefits**	2.8 (1.1)		0.06	0.44***	0.34***	0.46***	0.33***		
**Nurse satisfaction with given care**	5.6 (0.8)	0.91	0.05	0.33***	0.32***	0.30***	0.32***	0.24***	
**Stress symptoms**^**a**^	3.5 (0.7)	0.84	0.09	0.49***	0.32***	0.57***	0.36***	0.30***	0.17**

*Abbreviations*: *SD* standard deviation, *SE* structural empowerment, *W-PLB* work-personal life benefits, *NSC* nurse satisfaction with given care, α Cronbach’s Alpha values

*p < 0.05, **p < 0.01, ***p < 0.001

^a^higher scores represent fewer stress symptoms

**Table 2:** Structural empowerment, thriving, work-personal life benefits, job performance/nurse satisfaction with given care and stress symptoms for those with turnover intentions ‘No’ (*n* = 275; 67.2%) and ‘Yes’ (*n* = 131; 32.0%)

Turnover intentions	No *n* = 229–274 mean (SD)	Yes *n* = 114–131 mean (SD)	*P*-value	ES Cohen’s d Point estimate (95% CIs)
**Structural empowerment**	20.0 (3.5)	16.6 (3.3)	< 0.001	1.0 (0.7;1.2)
**Thriving**	5.6 (0.8)	4.5 (1.0)	< 0.001	1.3 (1.1;1.5)
• **Learning**	5.8 (0.8)	4.9 (1.1)	< 0.001	1.0 (0.8;1.2)
• **Vitality**	5.4 (0.9)	4.1 (1.1)	< 0.001	1.3 (1.1;1.6)
**Work-personal life benefits**	3.0 (1.1)	2.3 (0.9)	< 0.001	0.7 (0.5;0.9)
**Nurse satisfaction with given care**	5.6 (0.8)	5.5 (0.8)	0.165	0.2 (−0.1;0.4)
**Stress symptoms**^**a**^	3.7 (0.6)	3.1 (0.6)	< 0.001	1.0 (0.8;1.2)

Independent student t-test, when n does not add to 409 for the total sample, there are internal missing data,

*Abbreviations*: *SD* standard deviation, *ES* effect size, *CIs* confidence intervals

### H1 Thriving as a mediator between structural empowerment and work-personal life benefits

The model explained 18% of the variance in work-personal life benefits (Table 3, Model 2, *p* < 0.001). The relationship between structural empowerment and work-personal life benefits was positive, statistically significant in Model 1 (standardized coefficient 0.33, *p* < 0.001) (H1a) and non-significant in Model 2 when thriving was added 0.115, *p* = 0.069). The relationship was mediated through thriving (indirect effect 0.22; Boot SE 0.04; Boot 95% CIs 0.14 to 0.30) (H1b). Analysis without outliers in the model for work-personal life benefits showed that structural empowerment remained statistically significant in Model 2 when thriving was added 0.126, *p* = 0.044 (see Supplementary file 2).

**Table 3:** Relationships between structural empowerment, thriving and the outcomes work-personal life benefits, nurse satisfaction with given care (NSC), stress symptoms, and turnover intentions

	**Outcomes**				
	**Linear regression analyses**^**a**^			**Logistic regression analysis**	
	**Standardized Coefficient**			**Unstandardized Coefficient**^b^	**ExpB** (95%CI)^c^
	**Work-personal life benefits** *n* = 341	**NSC** *n* = 324	**Stress symptoms**^**d**^ *n* = 326	**Turnover intentions** *n* = 340	
**Model 1, *p*-value**^**e**^	< 0.001	< 0.001	< 0.001	< 0.001	
** Structural empowerment**	0.33***	0.32***	0.36***	−0.30***	0.74 (0.68;0.80)
** Age**			0.10*	−0.03*	0.97 (0.94;1.00)
*** R***^**2**^	0.11	0.10	0.14		
** Nagelkerke** ***R***^**2**^				0.26	
**Model 2, *p*-value**^**e**^	< 0.001	< 0.001	< 0.001	< 0.001	
** Structural empowerment**	0.12^ ns^	0.20**	0.10^ ns^	−0.17***	0.84 (0.76;0.93)
** Thriving**	0.35***	0.19**	0.41***	−1.06***	0.34 (0.24;0.50)
** Age**			0.10*	−0.04*	0.96 (0.93;0.99)
*** R***^**2**^	0.18	0.13	0.24		
** Nagelkerke** ***R***^**2**^				0.38	
** VIF**	1.6	1.7	1.7		
**Indirect effect, point estimate, (Boot SE) and Boot 95% CIs, 5000 boots**	0.22 (0.04) 0.14;0.30	0.12 (0.04) 0.05;0.20	0.26 (0.04) 0.17;0.35	−0.17 (0.03) −0.25;−0.12	

*Abbreviations: VIF *variance inflation factor,* SE *standard error,* CIs *confidence intervals

^a^Linear regression analyses using PROCESS, ^b^Logistic regression analyses using PROCESS and ^c^SPSS (to get OR Odds Ratio/ExpB, and estimates in Model 1 for turnover intentions). ^d^higher scores represent fewer stress symptoms, ^e^Model 1 includes structural empowerment (all outcomes) and age (outcome stress symptoms and turnover intention) as independent variables and in Model 2 thriving is added. SPSS was used to get VIF values, highest VIF presented

*p < 0.05, **p < 0.01, ***p < 0.001, ^ns^ non-significant

### H2 Thriving as a mediator between structural empowerment and job performance measured as nurse satisfaction with given care

The model explained 13% of the variance in nurse job performance (satisfaction with given care) (Table 3, Model 2, *p* < 0.001). The relationship between structural empowerment and job performance was positive (standardized coefficient 0.32, *p* < 0.001 Model 1 [H2a]; 0.20, *p* = 0.004 Model 2 when thriving was added), and the relationship was mediated through thriving (indirect effect 0.12; Boot SE 0.04; Boot 95% CIs 0.05 to 0.20) (H2b).

### H3 Thriving as a mediator between structural empowerment and work-related stress symptoms

The model explained 24% of the variance in stress symptoms (Table 3, Model 2, *p* < 0.001). The relationship between the variable structural empowerment and the variable stress symptoms was positive (standardized coefficient 0.36, *p* < 0.001 Model 1[H3a]; 0.10, *p* = 0.108 Model 2), and the relationship was mediated through thriving (indirect effect 0.26; Boot SE 0.04; Boot 95% CIs 0.17 to 0.35) (H3b). Note that higher scores on the stress symptoms factor represent fewer stress symptoms.

### H4 Thriving as a mediator between structural empowerment and turnover intentions

An increase in structural empowerment and an increase in thriving decreased the odds of being in the group with turnover intentions (OR structural empowerment 0.84; 95% CIs 0.76 to 0.93 [H4a]; and OR thriving 0.34; 95% CIs 0.24 to 0.50, *p* < 0.001 for both). Nagelkerke *R*^2^ for the model was 0.38 (*p* < 0.001 Model 2); the indirect effect of thriving was −0.17, boot SE 0.03 and Boot 95% CIs −0.25 to −0.12 (H4b).

## Discussion

The study is the first to examine and confirm thriving as a mediator between registered nurses’ structural empowerment and the outcomes work-personal life benefits (H1b), nurse satisfaction with given care (H2b), work-related stress (H3b), and turnover intentions (H4b). In line with our hypotheses, the results also showed that there were positive relationships between structural empowerment and work-personal life benefits (H1a) and satisfaction with given care (H2a). Furthermore, higher structural empowerment scores were related to fewer stress symptoms (H3a), and there was a negative relationship between structural empowerment and turnover intentions (H4a). The results are in line with Kanter’s theory of structural empowerment [13]. According to the theory, good access to structural empowerment, such as information, support, resources, and development opportunities as well as formal and informal empowerment, is related to improved staff wellbeing (c.f., our results of work-personal life benefits, fewer stress symptoms) and performance (c.f., our results of nurse satisfaction with given care).

Our results of thriving are also in line with Spreitzer’s model of thriving at work [1] where contextual factors are described as antecedents to thriving, c.f., structural empowerment in our study. Outcomes of thriving described in the theory are for example development and health, and reviews of thriving [2, 3] mention outcomes such as health, wellbeing, job performance and turnover intentions. In our study, the positive psychological state of thriving positively mediated the relationships between structural empowerment and staff job performance and work-personal life benefits. Previous research has also found thriving positively related to job performance [32, 38, 39], and in personal life positively related to life satisfaction [29]. Higher thriving was, in our study, also related to fewer negative outcomes such as stress symptoms (cf. Okros & Virga’s [53], study of mental health complaints) and turnover intentions cf. [29]. Among Swedish telephone nurses, both stress and turnover intentions have been described to be quite common [10, 11].

Furthermore, our results add to previous research on thriving as a mediator e.g., [38, 39]. Other studies, not from the healthcare sector, have shown that thriving mediates the relationship between, for example, co-workers’ social behaviours [38] and job performance [32, 38, 39]. In our results, thriving mediated the relationship between structural empowerment (which includes social aspects such as support from managers and colleagues as well as networks within and outside the organization) and job performance, measured as nurse satisfaction with given care. Furthermore, thriving has been found to be a mediator between contextual factors and outcomes such as job satisfaction [34], organizational commitment [40], and between workplace safety and wellbeing (measured as job satisfaction, physical and mental health complaints) [53]. These findings can be compared with our results showing that thriving is a mediator between empowering structures, and the two outcomes stress symptoms and turnover intentions due to job dissatisfaction. Researchers also describe thriving as a personal resource at work [53].

The variance explained by the variables, structural empowerment and thriving, in the models was 24% for stress symptoms, 18% for work-personal life benefits, and 13% for nurse satisfaction with given care. Thus, even if statistically significant, the explained variances were quite low, and thus the clinical relevance might be questioned. However, regarding turnover intentions, the Nagelkerke R^2^ for the model was 0.38, and the ES for thriving and structural empowerment when comparing the group with turnover intentions and the group without was large, indicating that the results of turnover intentions are of clinical relevance. Among telephone nurses, studies have found that they generally enjoy their work [11], feel that they learn a lot but need to work part-time due to a stressful job, and turnover is quite common [10, 11, 54]. Telephone nurses have also expressed concerns regarding patient safety due to stress based on organizational factors [55]. Thus, managers should promote good access to empowering structures for their telephone nurses and strive to increase thriving among nurses to prevent turnover intentions. This is also supported by a previous review [56] which showed that empowerment and thriving were related to lower staff turnover.

Our results from the bivariate correlation analyses showed that the two factors of thriving, i.e., learning and vitality, both correlated with all three outcomes, and the strength of the relationship was mostly medium effect size, except for the relationship between vitality and stress symptoms, where the effect size was large. The telephone nurses’ structural empowerment scores showed a moderate level, which is similar as in other studies of nurses e.g., [21, 23, 57]. Although telephone nursing has been reported as a stressful job in previous research [11], stress symptoms were not that common in our study. It may be that working part-time as described earlier [11] helped to manage work and reduce negative health impact. Working part-time shouldn’t be the preferred solution, instead more resources are needed. Turnover intentions were reported among one third of the telephone nurses and among those 52 had also taken action to find a new job. A quite high figure in an operation staffed entirely with nurses. The present results among Swedish telephone nurses emphasize the importance of empowering conditions (good access to information, support, opportunities and resources, a visible central job of importance with internal and external networks that facilitate work) for nurses’ thriving, job performance, work-personal life benefits, stress symptoms and turnover intentions. The links between nurses’ structural empowerment and their wellbeing and performance have been observed in several studies (for example [6, 22, 24, 58], whereas the link with thriving has been less explored within nursing. The results are also in line with several prerequisites emphasized in interviews with telephone nurses such as collegial support, development opportunities, possibility to influence work time and breaks, joint coffee and lunch breaks stimulating informal networks, regular feedback on performance, support from manager and technical support [10]. All being aspects similar to structural empowerment [13] and antecedents of thriving [2].

### Methodological considerations

Study limitations are the cross-sectional design, which does not allow causality conclusions, the response rate of 47.6%, which decreases generalizability, the use of self-reports only and the use of a one-item measure for the outcome work-personal life benefits. Furthermore, the use of a cross-sectional design also weakens the results from the mediation analyses [59]. For the thriving scale CFA indicated acceptable fit according to CFI but a mediocre fit according to RMSEA. Another weakness of the study is that the discriminant validity of the scales was not tested. The strengths are that all telephone nurses in Sweden were invited, and validated instruments were used. It should be noted that the data were collected in 2017. We consider the data to be of value because during the years of the Covid-19 pandemic there were very special conditions in telephone nursing and therefore several years that did not reflect the normal everyday life of telephone nurses, which our used data did. And a study inviting all telephone nurses in Sweden is not that common. In future research on thriving, it will be important to use a time lagged design, and to study the antecedents of thriving and outcomes among nurses in other settings, as telephone nurses’ work might be more central and visible compared to other settings where nurses work alongside other healthcare personnel. Furthermore, outcomes of staff reported structural empowerment, and thriving such as job performance could also be studied using for example first line managers’ assessments. And outcomes of work-personal life benefits could be measured using short, validated instruments instead of the use of a one-item measure.

## Conclusions

Thriving – a mutual sense of learning and vitality is a mediator in relationships between structural empowerment and both positive and negative working life outcomes such as job performance, work-personal life benefits, stress symptoms and turnover intentions. Managers should strive to improve nurses’ access to structural empowerment, which in turn relates to nurses’ thriving, reduces turnover intentions and improves quality of working life for nurses.

## Implications

To increase staff thriving – a sense of both learning and vitality – managers need to work in line with Kanter’s theory of structural empowerment [13] and improve telephone nurses’ access to opportunities to develop and advance at work, such as on-the-job training as well as formal education. In addition, good access to support from managers, colleagues and technical staff and good access to resources such as time and more staff at work allowing staff to also recover, exercise and socialize with colleagues during working time. Furthermore, good access to important information that facilitates the work, such as improvement of the computerized decision support system, up-to-date information on ongoing change processes and goals for the unit. Additionally, managers should make it easier for nurses to have informal networks that facilitate work, i.e., informal power. This is perhaps particularly important in telephone nursing as these nurses have a solitary job where they have phone calls with patients throughout the day and opportunities for breaks are few as they usually work against a queue of phone calls. Furthermore, nursing work is and should be highlighted as central and visible within the organization, i.e., formal power. In a previous study on telephone nurses, the work was perceived as both central to the organization and visible [11]. This is something that managers should strive for in order for telephone nurses to experience that their work is visible within the organization and valued as central and important. However, the last point concerning visibility invites the question: How visible is nurses’ work within healthcare organizations today in general? To decrease turnover intentions and keep nurses at the workplace, both good access to empowering structures and thriving seem to be of clinical importance.

## Supplementary Information

Supplementary Material 1.

Supplementary Material 2.

## Data Availability

The summary data are in the main document and Supplementary file 1 and 2. Research data (the data set with individual data) are not shared. Individual data are not available due to general data protection regulations (GDPR), and in line with the ethics application.
