# Supplementary material for: Thriving at work as a mediator between nurses’ structural empowerment and job performance, work-personal life benefits, stress symptoms and turnover intentions: a cross-sectional study
Source: BMC Nurs. 2025 Feb 14;24:175. doi: 10.1186/s12912-025-02828-0 (PMC11829515; doi:10.1186/s12912-025-02828-0)
Supplement: Supplementary file 1 — Supplementary Material 1. [file 12912_2025_2828_MOESM1_ESM.docx]

Supplementary file 1

**Table S1** Model fit summary

| Model Fit Summary | CFA  n=409 | Recommended values |
| --- | --- | --- |
| Chi-square | 161.77 |  |
| df | 33 |  |
| p-value | <0.001 | ns |
| CFI | 0.951 | 0.95^a^ |
| TLI | 0.918 | 0.95 ^a^, 0.90 ^b^ |
| RMSEA | 0.098 | 0.05; 0.08 to 0.10 ^c^ |
| RMSEA 90CIs | 0.083;0.113 |  |
|  |  |  |

Df degrees of freedom, CFI Comparative Fit Index, TLI Tucker-Lewis Index RMSEA Root Mean Square Error of Approximation, CI Confidence Interval, ns non-significant

^a^ Recommended cutoff close to 0.95 [50,51], ^b^ recommended ≥0.90 for NFI and TLI [52]. ^c^ RMSEA 0.05 good fit, 0.08 to 0.10 mediocre fit and >0.10 poor fit [51,53].

**Table S2** Standardized regression weights

| Items | Standardized regression weights |
| --- | --- |
| Learning |  |
| …continue to learn … | 0.823 |
| … not learning (reversed) | 0.353 |
| …developed a lot … | 0.708 |
| …learning often | 0.900 |
| …continually improving | 0.911 |
| Vitality |  |
| …alive and vital | 0.883 |
| …energy and spirit | 0.926 |
| … looking forward to each new day | 0.875 |
| … don’t feel very energetic (reversed) | 0.419 |
| …alert and awake | 0.808 |
