# Supplementary material for: Thriving at work as a mediator between nurses’ structural empowerment and job performance, work-personal life benefits, stress symptoms and turnover intentions: a cross-sectional study
Source: BMC Nurs. 2025 Feb 14;24:175. doi: 10.1186/s12912-025-02828-0 (PMC11829515; doi:10.1186/s12912-025-02828-0)
Supplement: Supplementary file 2 — Supplementary Material 2. [file 12912_2025_2828_MOESM2_ESM.docx]

Supplementary file 2

**Table S1.** Relationships between structural empowerment, thriving and the outcome work-personal life benefits, with and without outliers in the analyses

| Linear regression analyses^a^ | Outcome | | |
| --- | --- | --- | --- |
|  | **Standardized Coefficient** | | |
|  | **Work-personal life benefits** n=341 | **Work-personal life benefits without outliers** n=339 |  |
| Model 1, p-value^b^ | <0.001 | <0.001 |  |
| Structural empowerment | 0.33*** | 0.36*** |  |
| R^2^ | 0.11 | 0.13 |  |
| Nagelkerke R^2^ |  |  |  |
| Model 2, p-value^e^ | <0.001 | <0.001 |  |
| Structural empowerment | 0.115^ns^ | 0.126* |  |
| Thriving | 0.35*** | 0.37*** |  |
| R^2^ | 0.18 | 0.21 |  |
| Nagelkerke R^2^ |  |  |  |
|  |  |  |  |
| Indirect effect,  point estimate, (Boot SE) and Boot 95% CIs, 5000 boots | 0.22  (0.04)  0.14;0.30 | 0.23  (0.04)  0.16;0.30 |  |

Abbreviations

SE standard error, CIs confidence intervals,

^a^ Linear regression analyses using PROCESS, ^b^Model 1 includes structural empowerment as independent variables and in Model 2 thriving is added. *p<0.05, **p<0.01, ***p<0.001, ^ns^ non-sinificant
